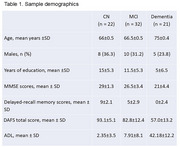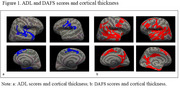# Self versus proxy‐evaluated functionality: which has a stronger relation with white and gray integrity in patients with Alzheimer's Disease

**DOI:** 10.1002/alz70856_107247

**Published:** 2026-01-08

**Authors:** Renata Kochhann, Maila Rossato Holz, Marcia L. Chaves, Rochele Paz Fonseca, Wyllians Vendramini Borelli

**Affiliations:** ^1^ Hospital Moinhos de Vento, Porto Alegre, Rio Grande do Sul, Brazil; ^2^ PUCRS, Porto Alegre, Rio Grande do Sul, Brazil; ^3^ Hospital de Clinicas de Porto Alegre, Porto Alegre, RS, Brazil; ^4^ UFMG, Belo Horizonte, Minas Gerais, Brazil; ^5^ Universidade Federal do Rio Grande do Sul, Porto Alegre, Rio Grande do Sul, Brazil; ^6^ Centro de Memória, Hospital Moinhos de Vento, Porto Alegre, RS, Brazil

## Abstract

**Background:**

Functional ability is one of the main determinants of dementia diagnosis and is traditionally evaluated by caregiver/proxy reporting. However, as many elderly are living alone, healthcare providers often need to rely on self‐evaluation. Therefore, we aimed to compare self‐ and proxy‐evaluated functional ability and to determine its cerebral white and gray matter correlates.

**Methods:**

Individuals attending a tertiary memory clinic in Brazil were invited to participate. They underwent a clinical, cognitive, and MRI assessment during the same day. Community‐dwelling controls were also recruited. All participants completed the Direct Assessment Functional Status (DAFS) and their informants responded to the Activities of Daily Living Questionnaire (ADL). Cortical thickness was evaluated by analyzing volumetric T1‐scans using FreeSurfer v7.4.0. Statistical analysis was performed using a Generalized Linear Model (GLM) adjusting for age.

**Results:**

Twenty‐one dementia patients, 32 Mild Cognitive Impairment (MCI) and 22 control participants were included. Dementia group showed worse MMSE (*p* < 0.001), and functional abilities evaluated by DAFS and ADL when compared with MCI and Controls (*p* < 0.001, Tab. 1). Volumetric analyses in the whole sample demonstrated significant negative associations between ADL scores and cortical thickness of frontal, parietal and temporal lobes (Figure 1a‐d, *p* < 0.05). Cortical thickness across all lobes was positively associated with DAFS scores (Figure 2a‐d, *p* < 0.05).

**Conclusions:**

Functional independence measured by ADL were associated with frontal and parietal cortical thickness, while DAFS scores demonstrated a more global evaluation of cortical atrophy. Therefore, self‐evaluation of functional abilities appears to be a stronger marker of cortical thickness than a proxy‐reported measure.